# TheiaEuk: a species-agnostic bioinformatics workflow for fungal genomic characterization

**DOI:** 10.3389/fpubh.2023.1198213

**Published:** 2023-08-01

**Authors:** Frank J. Ambrosio, Michelle R. Scribner, Sage M. Wright, James R. Otieno, Emma L. Doughty, Andrew Gorzalski, Danielle Denise Siao, Steve Killian, Chi Hua, Emily Schneider, Michael Tran, Vici Varghese, Kevin G. Libuit, Mark Pandori, Joel R. Sevinsky, David Hess

**Affiliations:** ^1^Theiagen Genomics, Highlands Ranch, CO, United States; ^2^Nevada State Public Health Laboratory, Reno, NV, United States; ^3^Alameda County Public Health Laboratory, Oakland, CA, United States; ^4^Public Health Laboratories, Division of Disease Control and Health Statistics, Washington State Department of Health, Shoreline, WA, United States; ^5^Department of Pathology and Laboratory Medicine, Reno School of Medicine, University of Nevada, Reno, NV, United States; ^6^Department of Microbiology and Immunology, Reno School of Medicine, University of Nevada, Reno, NV, United States

**Keywords:** *Candida auris*, epidemiology, whole-genome sequencing, bioinformatics, emerging pathogens

## Abstract

**Introduction:**

The clinical incidence of antimicrobial-resistant fungal infections has dramatically increased in recent years. Certain fungal pathogens colonize various body cavities, leading to life-threatening bloodstream infections. However, the identification and characterization of fungal isolates in laboratories remain a significant diagnostic challenge in medicine and public health. Whole-genome sequencing provides an unbiased and uniform identification pipeline for fungal pathogens but most bioinformatic analysis pipelines focus on prokaryotic species. To this end, TheiaEuk_Illumina_PE_PHB (TheiaEuk) was designed to focus on genomic analysis specialized to fungal pathogens.

**Methods:**

TheiaEuk was designed using containerized components and written in the workflow description language (WDL) to facilitate deployment on the cloud-based open bioinformatics platform Terra. This species-agnostic workflow enables the analysis of fungal genomes without requiring coding, thereby reducing the entry barrier for laboratory scientists. To demonstrate the usefulness of this pipeline, an ongoing outbreak of *C. auris* in southern Nevada was investigated. We performed whole-genome sequence analysis of 752 new *C. auris* isolates from this outbreak. Furthermore, TheiaEuk was utilized to observe the accumulation of mutations in the FKS1 gene over the course of the outbreak, highlighting the utility of TheiaEuk as a monitor of emerging public health threats when combined with whole-genome sequencing surveillance of fungal pathogens.

**Results:**

A primary result of this work is a curated fungal database containing 5,667 unique genomes representing 245 species. TheiaEuk also incorporates taxon-specific submodules for specific species, including clade-typing for *Candida auris (C. auris)*. In addition, for several fungal species, it performs dynamic reference genome selection and variant calling, reporting mutations found in genes currently associated with antifungal resistance (*FKS1*, *ERG11*, *FUR1*). Using genome assemblies from the ATCC Mycology collection, the taxonomic identification module used by TheiaEuk correctly assigned genomes to the species level in 126/135 (93.3%) instances and to the genus level in 131/135 (97%) of instances, and provided zero false calls. Application of TheiaEuk to actual specimens obtained in the course of work at a local public health laboratory resulted in 13/15 (86.7%) correct calls at the species level, with 2/15 called at the genus level. It made zero incorrect calls. TheiaEuk accurately assessed clade type of *Candida auris* in 297/302 (98.3%) of instances.

**Discussion:**

TheiaEuk demonstrated effectiveness in identifying fungal species from whole genome sequence. It further showed accuracy in both clade-typing of *C. auris* and in the identification of mutations known to associate with drug resistance in that organism.

## 1. Introduction

Microbial fungal pathogens are a major public health concern estimated to affect over 13 million patients annually, with mortality of over 1 million patients annually (1, 2). Fungal infections are especially problematic for patients with conditions such as HIV/AIDs, chronic obstructive pulmonary disease (COPD), asthma, tuberculosis and patients undergoing cancer treatments. Fungal pathogens remain understudied compared to prokaryotic pathogens and often present difficulties in identification and characterization (3–8).

Antifungal drugs are the primary treatment for pathogenic fungal infections. There are four major classes of antifungal drugs: echinocandins (caspofungin), azoles (fluconazole), polyenes (amphotericin B), and the pyrimidine analogue 5-flucytosine. However, the overuse and misuse of these drugs have led to the emergence of drug-resistant strains of these fungi and increasingly prevalent multi-drug resistant fungal infections (9–11). Given the limited classes of drugs to treat fungal infections, the threat of multidrug resistant fungal infections poses a public health menace (9, 11). These strains are often more difficult to treat, resulting in longer hospital stays, higher healthcare costs, and increased mortality rates. In fact, some studies have shown that mortality rates can be as high as 50% in patients with drug-resistant *Candida albicans* infections (9, 10).

*Candida auris* is a fungal pathogen that has rapidly emerged as a public health concern. It was originally identified in Japan in 2009, and has since been found in over 30 countries, including the United States (10, 12–14). This organism is particularly concerning because it has demonstrated resistance to multiple antifungal drugs, making treatment of infections challenging. In a study of *C. auris* isolates from multiple continents, fluconazole resistance was detected in 93% of isolates, amphotericin B resistance was detected in 35%, and echinocandin resistance was detected in 7% (13). The scope of antifungal treatment options is limited, making managing infections with *C. auris* difficult (1). The ability to resist treatment combined with the ability to cause invasive infections in patients who are already ill and weakened leads to high *C. auris* mortality (13, 15). This highlights the need for enhanced surveillance methods that detect not only the presence of *C. auris,* but also whether the isolate is part of an ongoing outbreak and what antifungal resistance determinants the isolate may harbor.

While there are numerous other fungal pathogens of public health concern, certain species exist as growing antimicrobial resistance threats. *Aspergillus fumigatus* is a common opportunistic airborne fungal pathogen that can cause serious infections in humans. Resistance to several antifungal drugs, including azoles, has been observed in this fungus (16, 17). *Cryptococcus neoformans* is a fungal pathogen that causes serious infections in individuals with weakened immune systems, and often presents difficulties in infection management due to resistance to several antifungal drugs (18, 19). *C. albicans* is a type of fungus commonly found on the skin and mucous membranes of humans. Although often harmless, it can cause infections in vulnerable individuals, such as those with weakened immune systems, surgical wounds, or indwelling medical devices. In recent years, *C. albicans* has also become a growing public health threat due to its increasing resistance to antifungal drugs (20, 21).

Genomic sequencing is a useful tool for analyzing fungal pathogens for public health investigations (22). By analyzing individual pathogen genomes, researchers can identify the species responsible for a patient infection, sub-type the organism, and detect mutations that are associated with resistance to antifungal medicines. For this to be realized, accessible and easy-to-use bioinformatic pipelines for genomic fungal analysis must be developed and deployed to the public health community. To this end, we developed TheiaEuk, a pipeline that performs genome assembly and taxonomic identification of 245 fungal species across 138 genera from FASTQ files generated by whole-genome sequencing. Following taxonomic identification, species-specific analyses are automatically launched. For example, when *C. auris* is detected, clade designation and mutations that are likely to result in antifungal resistance are automatically reported. Lastly, genome assemblies produced by the TheiaEuk pipeline are compatible with several tools for downstream phylogenetic analysis especially when accessed in the Terra platform (23). We demonstrate that the TheiaEuk pipeline provides the bioinformatic tools needed by public health and medical professionals to utilize whole-genome sequencing to characterize and to phylogenetically assess fungal pathogens.

## 2. Materials and methods

### 2.1. TheiaEuk pipeline

#### 2.1.1. TheiaEuk implementation

**Figure 1:**
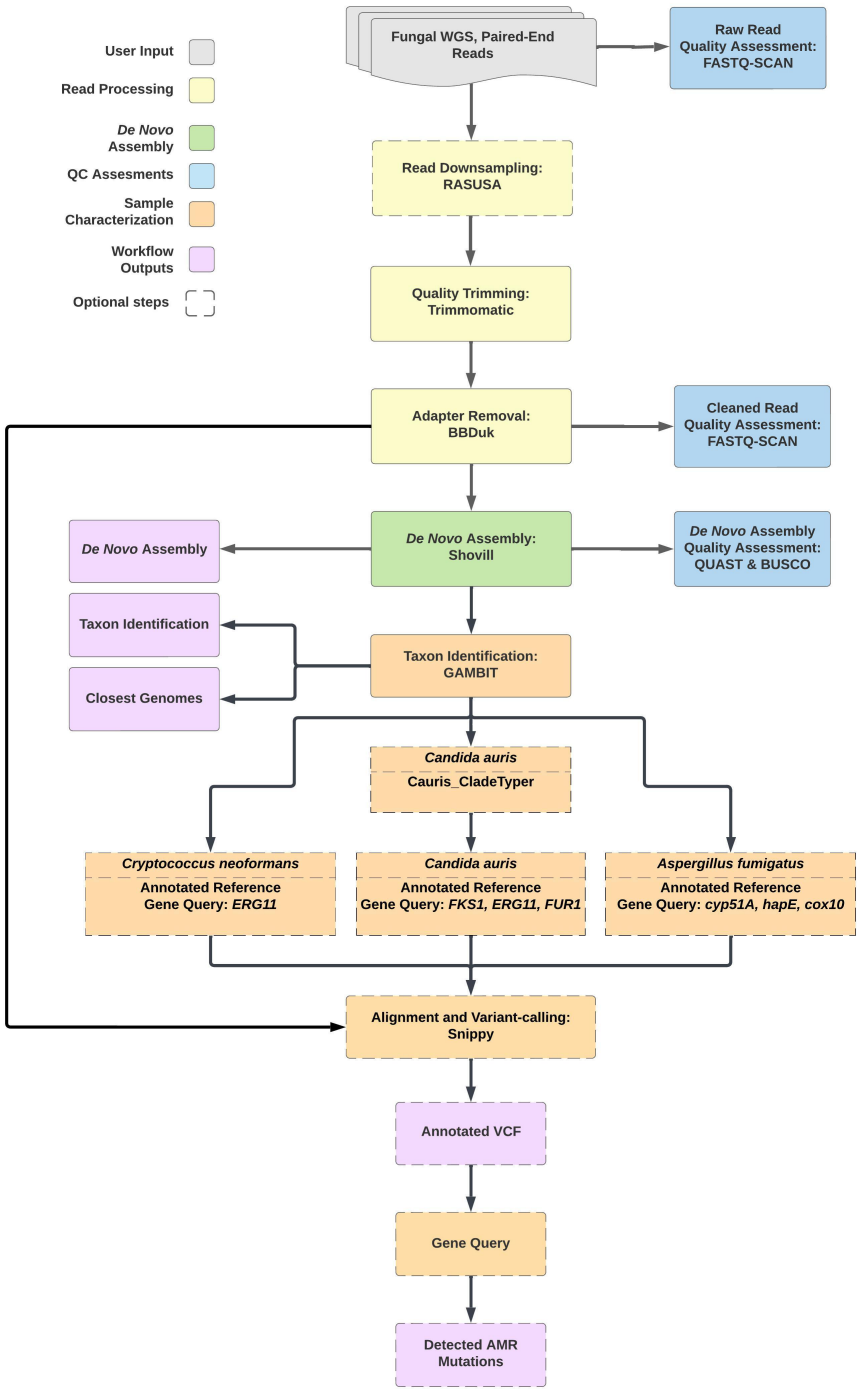
The TheiaEuk workflow is a species-agnostic bioinformatics pipeline for fungal genome characterization. Input FASTQ files from WGS of fungal pathogens are assessed for quality and *de novo* assembled regardless of species. Taxonomic identification is performed by GAMBIT using a custom FIGURE 1 (Continued)fungal database. Taxa-specific sub-workflows for *Candida auris*, *Cryptococcus neoformans*, and *Aspergillus fumigatus* proceed automatically based on the GAMBIT result.

The TheiaEuk workflow was designed to perform *de novo* genome assembly, quality assessment, and genomic characterization of fungal pathogen genomes from paired-end short read sequencing data (see text footnote 1). The workflow is written in the workflow description language (WDL) and as such may be implemented on the browser-based Terra platform (23, 24). The workflow can also be executed from the command line interface using WDL workflow engines such as Cromwell or miniWDL (25, 26). TheiaEuk will process and analyze Illumina paired-end FASTQ inputs using default parameters established for robust fungal pathogen analysis; these parameters can be modified by users from within the graphical user interface of Terra. The workflow utilizes many existing bioinformatics tools as cited in the sections below and produces outputs with industry standard file formats to facilitate downstream analyses. Comparison of TheiaEuk to other pipelines that have been deployed for fungal genome analysis, MycoSNP (27) and Nullarbor (28), was presented in Gorzalski et al. (29). The structure of the pipeline is described below and illustrated in Figure 1.

#### 2.1.2. Read trimming and quality control

To avoid errant characterization from poor sequencing data, TheiaEuk performs raw read screening on input FASTQ files to determine whether the workflow will proceed to subsequent analysis or be halted in the event of scarce or problematic input data. This step assesses the number of base pairs, number of reads, and proportion of reads in each input FASTQ file. It also employs MASH sketches to estimate genome size and sequencing depth (30). Samples that pass the initial read screen proceed to an optional step in which reads are randomly subsampled to 150× read depth using RASUSA to conserve computational resources (31). Next, TheiaEuk performs read trimming using Trimmomatic and adapter trimming using BBDuk (32, 33). Read trimming is followed by an additional read screening step to determine if the sequencing data still passes the screening parameters. Samples which meet the parameters proceed automatically to genome assembly.

#### 2.1.3. Genome assembly

TheiaEuk performs *de novo* genome assembly using the Shovill package (34). Shovill is a software package containing several assembly algorithms commonly used for bacterial genome assembly including SPAdes (35) and SKESA (36). SKESA has been set as the default assembler, but the ability to select an alternative assembly program is made available to the user. All the assembly programs within Shovill are designed to assemble haploid genomes, which limits the scope of the pipeline to fungal pathogens with single copies of unpaired chromosomes. Certain downstream modules, particularly GAMBIT due to its *k*-mer-based approach, may be robust to bioinformatics challenges associated with *de novo* assembly of diploid organisms. Nonetheless, these assemblies may be highly fragmented or error prone. Results for diploid organisms must at minimum be assessed with caution and in conjunction with the level of heterozygosity. Following *de novo* assembly, TheiaEuk performs quality assessment of the assembly using QUAST and BUSCO (37, 38).

#### 2.1.4. Taxonomic identification

Following genome assembly, the assembly FASTA files are passed to the Genomic Approximation Method for Bacterial Identification and Tracking (GAMBIT) tool for taxonomic identification (39). GAMBIT infers taxonomy by querying a sample genome against a database of genomes with known taxonomic information and identifying the most similar genome to the query. If the distance between the query genome and the closest genome is within a built-in species threshold, GAMBIT reports the species of the closest genome as the predicted species for the query genome. If not, GAMBIT determines if the query is close enough to be considered a member of the closest genome’s genus, otherwise it will not make a taxonomic prediction for the query genome.

Within TheiaEuk, GAMBIT is implemented with the default parameters (*k* = 11 and prefix = ATGAC), and the taxon predicted by GAMBIT is reported as well as the ten closest genomes within the GAMBIT database to the query sample. The only previously published GAMBIT database is exclusive to prokaryotic species, therefore we developed a novel fungal database for identification of fungal pathogens, as described below. This novel fungal database is used by default within TheiaEuk.

#### 2.1.5. Taxa-specific modules (clade typing)

Based on the taxonomic identification made by GAMBIT, TheiaEuk proceeds with taxa-specific modules. For samples identified as *C. auris*, TheiaEuk will perform clade typing using GAMBIT with a custom database consisting of five reference genomes representing the five major clades of *C. auris* (Supplementary Table S1). GAMBIT reports the reference genome that is most similar to the query genome and the associated clade is reported for the sample.

#### 2.1.6. Taxa-specific modules (AMR determinant detection)

For samples identified as *C. auris*, *A. fumigatus*, and *C. neoformans*, TheiaEuk invokes a module which aligns input FASTQ files to a species-appropriate annotated reference genome using Snippy (40). To detect potential antimicrobial resistance determinants, the resulting VCF files may be queried for gene and product names that are associated with antimicrobial resistance following TheiaEuk analysis. Snippy has been used previously to detect mutations in the *FKS1* gene of *Candida* species (41). For *C. auris*, the antimicrobial resistance detection module aligns reads to a clade-specific reference genome and automatically queries the resulting VCF files for three genes associated with antimicrobial resistance (*FKS1*, *ERG11*, *FUR1*). A list of all mutations that have been detected in these select genes are reported to the user. The reference genomes for each *C. auris* clade are indicated in Supplementary Table S1.

### 2.2. Fungal GAMBIT database creation

In order to infer taxonomic assignments from fungal genomic data, we created a novel fungal GAMBIT database using a similar process as the prokaryotic GAMBIT database (39). The process of creating a GAMBIT database requires the calculation of compressed representations of each genome that will be included in the database, or GAMBIT signatures, which enable the calculation of GAMBIT distances between genomes. In order for GAMBIT to generate a species assignment for a query genome, the distance between the query genome and the closest genome within the database must be below the maximum distance between genomes within that species (species diameter). As such, the GAMBIT database must be curated to ensure that species diameters are non-overlapping and unbiased by mislabeled or poor-quality genomes.

The novel fungal database was created by downloading all the fungal genomes available on GenBank as of 2022-11-30 and curating this list of genomes to exclude poorly represented species and mislabeled genomes. GAMBIT signatures were computed using the same criteria as the most recent GAMBIT bacterial database (*k* = 11 and prefix = ATGAC). For inclusion in the database, species were required to have at least two genomes in GenBank and at least one genome representing the species in RefSeq (42). Subsequently, we curated the database on the basis of the species diameter. Specifically, we computed the GAMBIT diameter of each species and excluded species with either (i) a diameter of zero or (ii) a combination of three or fewer genomes and a diameter greater than 0.75. The database was also manually curated to remove genomes which were clearly highly distant from all other genomes within the species, as these were likely mislabeled on submission.

To establish a set of genomes with non-overlapping species diameters, it was necessary to divide nine species into subspecies groups. In the event that the closest genome in the database to a query genome is a member of a subspecies, GAMBIT will report the parent species as the taxonomic assignment. In addition, two pairs of species were too closely related to distinguish (*Aspergillus flavus/Aspergillus oryzae* and *Aspergillus niger/Aspergillus welwitschiae*), therefore were combined. If the distance between a query genome and the closest genome in the GAMBIT database is greater than the species diameter, GAMBIT checks if the sample is within the genus diameter and attempts to report a genus for the genome. Genus diameters were computed similarly to species diameters, but were additionally curated by lowering the diameter to 95% of the minimum distance between the genus and other genera in the database and to 20% greater than the maximum species diameter of any species within the genus.

Ultimately, 245 fungal species from 138 genera are represented in the fungal database from a total of 5,667 fungal genomes. A table indicating the number of genomes and species diameter for each species represented in the database is indicated in Supplementary Table S2.

### 2.3. Fungal GAMBIT database validation

#### 2.3.1. GAMBIT versus ANI analysis

Analysis of GAMBIT distances versus average nucleotide identity (ANI) was performed using the GAMBIT distance values computed during the creation of the fungal database for all of the genomes in set 1 and set 2 (*k* = 11 and prefix = ATGAC). Set 1 included all *Candida* genomes within the fungal GAMBIT fungal database and set 2 included a diverse set of genomes across multiple genera. ANI was computed using FastANI (version 1.33) with default parameter values (*k*-mer size 16 and fragment length 3,000) (43). Pairwise comparisons were included in both the statistical analysis and visualizations if the percent of mapped fragments was at least 50%. Figures were generated using scripts adapted from Lumpe et al. using Matplotlib (44, 45).

#### 2.3.2. ATCC mycology genomes

Validation of the fungal GAMBIT database using the ATCC Mycology Collection genomes was performed using the Gambit_Query workflow developed by Theiagen Genomics on Terra.^2^ All available fungal genomes were downloaded from the ATCC genome portal on 2023-03-08 (46–48). ATCC genomes downloaded from the ATCC genome portal were used exclusively for testing and were not included in the GAMBIT fungal database. GAMBIT was run with default parameters and we examined the predicted taxon and predicted taxon rank for agreement with the taxonomic annotation from ATCC.

#### 2.3.3. Sequenced isolates from Alameda County

In order to generate a diverse set of fungal genomes for assessing the accuracy of GAMBIT using the fungal database, 19 fungal samples from 18 distinct species were obtained from the Alameda County Public Health Laboratory. Whole genome sequencing of these fungal specimens was performed by the Nevada State Public Health Laboratory through an identical protocol as described below for sequencing of *C. auris* isolates from southern Nevada. The TheiaEuk workflow v1.0.0 was used to run GAMBIT with default parameters on Terra and we compared the predicted taxon from GAMBIT to the taxonomic assignment made using molecular techniques. Whole genome sequencing data for each specimen was submitted to NCBI’s Sequencing Read Archive (SRA); accessions are available in Supplementary Table S3.

### 2.4. Clade typing validation

Within the TheiaEuk pipeline, clade typing of *C. auris* is performed when a sample is predicted to be *C. auris* by GAMBIT. We tested the accuracy of the TheiaEuk clade typing module by querying 302 samples from a published *C. auris* dataset in which clades were assigned (49). Genomes in this dataset were originally derived from multiple studies, with clade type reported by Chow et al. (13, 49–54). Sequencing read data was pulled from NCBI’s SRA using the Theiagen Genomics SRA_Fetch workflow^3^ and analyzed using TheiaEuk v1.0.0 with default parameters.

### 2.5. Antimicrobial resistance mutation detection validation

To verify that TheiaEuk reports mutations in antimicrobial resistance genes in samples with known resistance determinants, we identified whole genome sequencing data for 219 *C. auris* samples from published datasets (55–57). FASTQ files for these samples were imported into Terra using the SRA_Fetch workflow and analyzed using TheiaEuk v1.0.0 with the default parameters. The outcome of the TheiaEuk AMR mutation detection module was compared to the known *FKS1* and *ERG11* mutations within each sample.

### 2.6. Southern Nevada *Candida auris* outbreak

#### 2.6.1. Specimen collection

*C. auris* specimens from an ongoing outbreak in southern Nevada were isolated from clinical samples collected from April 2022 to February 2023. Genomic data from 752 specimens is reported for the first time in this study, but several analyses utilize all sequenced isolates from the southern Nevada outbreak including an additional 209 specimens reported in Gorzalski et al. (29).

#### 2.6.2. Whole genome sequencing

Genomic DNA for sequencing was extracted using a combination of bead-beating (FastPrep-24, MP Biomedicals, Irvine, CA) and magnetic-bead purification (Maxwell RSC 48, Promega, Madison, WI). First, isolates were picked from Sabouraud Dextrose agar plates and mixed with silica beads (Lysing Matrix C, MP Biomedical). Cells were mechanically sheared with 2 cycles at 6.0 m/s for 30 s with a 5 min pause between (FastPrep-24, MP Biomedical). Genomic DNA was isolated using the PureFood Pathogen Kit (Promega) on a Maxwell RSC 48 (Promega) using the manufacturer’s protocol. Genomic DNA libraries were prepared using DNA Prep Kit (Illumina, San Diego, CA) using the manufacturer’s recommended protocol using a STARlet automated liquid handler (Hamilton Company, Reno, NV). Paired-end sequencing (2× 151) was performed using Illumina’s MiniSeq and NovaSeq 6000 to a minimum depth of 35× average coverage. Whole genome sequencing data for these specimens was submitted to NCBI’s sequencing read archive (SRA) and accessions are available in Supplementary Table S4. Samples were analyzed using the TheiaEuk workflow v1.0.0 with default parameters on Terra. Analysis of clade assignments and FKS1 mutations among these samples and an additional 209 specimens reported in Gorzalski et al. (29) was visualized using R and RStudio with the tidyverse package (58–60). Twelve samples with either assembly lengths greater than 14 Mbp or BUSCO completeness scores less than 90% were excluded from this analysis as noted in Supplementary Table S4.

#### 2.6.3. Antimicrobial susceptibility testing

*C. auris* antimicrobial susceptibility testing (AST) was performed using microbroth dilution and predefined gradient of antibiotic concentrations (Etest) methods. A patient isolate was grown on SabDex agar plate and incubated at 30°C in ambient air for 24 h and used to make 0.5 McFarland inoculum suspension in demineralized sterile water. The 0.5 McFarland suspension was measured by spectrophotometer to verify the 0.5 McFarland (80%–82% transmittance). Twenty microliters of 0.5 McFarland suspension were added into 11 mL of RPMI broth tube and 100 μL of the RPMI diluted sample was distributed to each well of a 96-well plate pre-loaded with antibiotics, then incubated along with control plates for 24 h at 35 °C. The same 0.5 McFarland inoculum suspension was used to inoculate a RPMI agar plate using a sterile cotton swab. A single Amphotericin B Etest strip was applied to middle of the agar surface using sterile forceps and incubated along with control plates for 24 h at 35 °C. The AST of the microbroth dilution panel was read using a parabolic magnifying mirror to determine the MIC (lowest concentration where there is ≤50% growth compared to growth control well). For the Amphotericin B Etest, MIC was interpreted at a value where there is 100% growth inhibition (number above where the ellipse intercepts Etest strip).

## 3. Results

### 3.1. TheiaEuk workflow

In response to an ongoing outbreak of *C. auris* in southern Nevada, Theiagen Genomics and the Nevada State Public Health Laboratory collaborated to develop a bioinformatics pipeline for analyzing *C. auris* WGS data: TheiaEuk. TheiaEuk is a species-agnostic workflow for fungal genome characterization that can be implemented through a graphical user interface using Terra. Briefly, this pipeline quality trims and assesses input paired-end short read sequencing data then creates a *de novo* assembly using the SKESA assembler (Figure 1) (36). Using the genome assembly, species taxon identification is performed by the Genomic Approximation Method for Bacterial Identification and Tracking (GAMBIT) tool. GAMBIT implementation in TheiaEuk uses a novel, curated fungal database containing 5,667 genomes and 245 species. For certain identified taxa, taxa-specific workflows are activated, such as a *C. auris* clade-typing tool and antifungal resistance detection.

### 3.2. Fungal GAMBIT database validation

GAMBIT was designed for microbial taxonomic identification by querying genome assemblies against a database and assigning taxonomy based on curated diagnostic thresholds (39). The initial GAMBIT database contained only prokaryotic genomes, but nothing precluded the extension of GAMBIT to eukaryotic microbes. Here we describe the development and validation of a novel fungal microbial database using the core GAMBIT logic.

**Figure 2:**
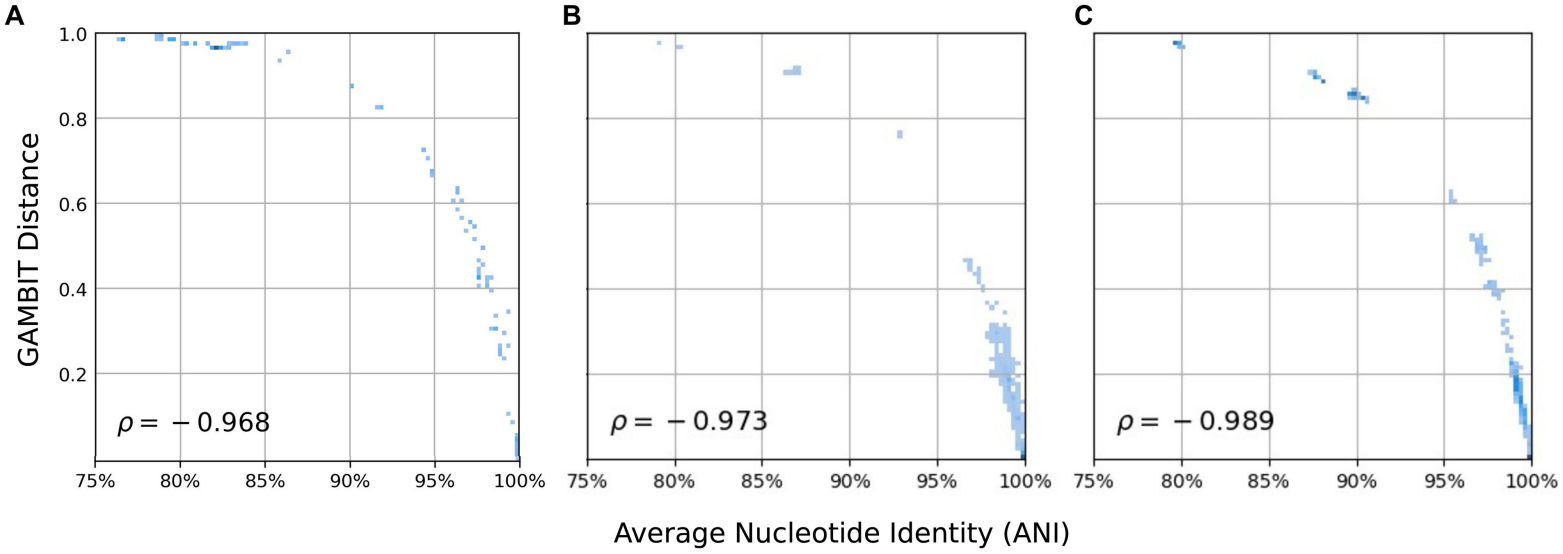
Relationship between GAMBIT distance and average nucleotide identity (ANI) for every pair of genomes within three datasets. **(A)** Image is reproduced from (39) and consists of 70 prokaryotic genomes from diverse taxa. This set is shown here to visualize the trend observed in prokaryotic genomes. **(B)** Set 1 consists of 381 *Candida* genomes (Spearman correlation = 97.3%) and **(C)** and set 2 consists of 126 fungal genomes from diverse species (Spearman correlation = 98.9%). Only pairwise comparisons where FastANI reported that greater than 50% of fragments mapped were included in statistical analyses and plotted.

First we demonstrate that eukaryotic microbial isolates have the same relationship as prokaryotes when comparing average nucleotide identity (ANI) versus GAMBIT distance (Figure 2) (39, 43). To this end, two sets of genomes were selected within the fungal database and ANI and GAMBIT distance computations were performed between every pair of genomes within each dataset. These fungal genomes demonstrate the same logarithmic relationship between ANI and GAMBIT distance as prokaryotic genomes (Figure 2A) which suggests that there is no difference between prokaryotic and eukaryotic microbes in terms of identification via GAMBIT. In the first dataset, we examined 318 genomes from the *Candida* genus (Figure 2B). For comparisons where FastANI reported an ANI value and the percent of mapped fragments was greater than 50% (13,389 genome pairs, 26.4% of comparisons), GAMBIT distance and ANI exhibited a Spearman correlation of 97.3%. This analysis was extended to a broader range of eukaryotic microbial species and demonstrated the same relationship with a Spearman correlation of 98.9% for pairwise comparisons where ANI values were reported (970 genome pairs, 12.3% of comparisons) (Figure 2C).

### 3.3. Validation of the fungal GAMBIT database using ATCC genomes

To assess the accuracy of the fungal GAMBIT database, the taxonomic assignments were validated using two sets of genomes with known taxonomic assignments. The first validation was performed using fungal genomes from the ATCC Mycology collection (46, 47). This dataset was selected due to the high level of confidence in the taxonomic assignment of these genomes and includes 190 fungal genomes from 61 genera and 109 species. In total, 135 of the genomes are represented at the species level within the GAMBIT database, 33 are represented only at the genus level, and 22 are not represented at the genus or species level. Of the genomes for which a species prediction was possible (135 genomes), GAMBIT reported the correct species for 126 genomes (Table 1). For the remaining 9 genomes, GAMBIT predicted either the correct genus (5 genomes) or made no taxonomic prediction (4 genomes). For the genomes that were represented only at the genus level (33 genomes), GAMBIT reported the correct genus for 3 genomes and reported no taxonomic assignment for 30 genomes. Finally, for the genomes that were not represented at the genus or species level within the GAMBIT database (22 genomes), GAMBIT made no taxonomic predictions, as expected. GAMBIT taxonomic assignment for each genome is indicated in Supplementary Table S5.

**Table 1:** Fungal GAMBIT database validation using ATCC Mycology genome collection.

		Expected assignment (ATCC)			Total
		Species	Genus	No assignment	
Observed assignment (GAMBIT)	Species	126	0	0	126
	Genus	5	3	0	8
	No assignment	4	30	22	56
	Total	135	33	22	190 Total Genomes

One hundred ninety fungal genomes were analyzed using GAMBIT with the fungal GAMBIT database. Based on the content of the fungal GAMBIT database, query genomes were expected to be identified at the species level, genus level, or not assigned. Correct taxonomic assignments made by GAMBIT for each possible taxonomic rank are shown. GAMBIT made no incorrect taxonomic assignments.

The data demonstrated that using the developed fungal database, GAMBIT reported either an accurate taxonomic assignment or no taxonomic assignment for all 190 genomes examined. Given the underrepresentation of high-quality fungal genomes in public repositories, the GAMBIT database is designed to perform taxonomic identification conservatively. Consequently, the majority of taxonomic assignments were at the lowest possible taxonomic rank (126/135 possible species assignments, 3/33 genus assignments), but 39 genomes were assigned to either a higher taxonomic rank or received no taxonomic assignment.

### 3.4. Validation of the fungal GAMBIT database using sequenced samples

Given the relative scarcity of fungal genomes available for validating the fungal GAMBIT database, the Nevada State Public Health Laboratory obtained 19 fungal samples from the Alameda County Public Health Laboratory and subjected them to whole genome sequencing. The samples represented 18 distinct fungal species according to previous reference laboratory biochemical and molecular laboratory techniques including *Aspergillus, Candida, Clavispora, Coccidioides, Cryptococcus, Kluyveromyces, Pichia, Trichophyton*, and *Yarrowia* species (Table 2). Sequencing data was analyzed using TheiaEuk with the fungal GAMBIT database to assess the accuracy of GAMBIT taxonomic identification. One sample did not produce quality sequencing data for successful completion of TheiaEuk (*A. flavus*). Of the remaining 17 species, 14 were represented at the species level within the fungal GAMBIT database, 2 at the genus level only (*Fusarium* of undetermined species and *Candida metapsilosis*), and 1 was not represented (*Trichophyton mentagrophytes*). Of the 15 samples where species assignments were possible, 13 were identified correctly at the species level and 2 were identified correctly at the genus level. Of the 2 samples where genus-level only assignments were possible, 1 was assigned the correct genus and 1 received no assignment. The sample that was not represented in the database received no assignment, as expected. Therefore, both validations of the fungal GAMBIT database demonstrated exclusively accurate taxonomic assignments, often at the lowest taxonomic level possible.

**Table 2:** Fungal GAMBIT database validation using genomes obtained from the Alameda County Public Health Laboratory and sequenced by the Nevada State Public Health Laboratory.

NCBI organism name	Expected GAMBIT genus assignment	Expected gambit species assignment	Observed GAMBIT genus assignment	Observed gambit species assignment	Identification method or isolate source
*Aspergillus terreus*	*Aspergillus*	*terreus*	*Aspergillus*	*terreus*	MALDI-TOF at MDL
*Candida albicans*	*Candida*	*albicans*	*Candida*	*albicans*	ATCC 14053
*Candida auris*	*Candida*	*auris*	*Candida*	*auris*	CDC B11903
*Candida dubliniensis*	*Candida*	*dubliniensis*	*Candida*	NA	Unknown
*Candida glabrata*	*Candida*	*glabrata*	*Candida*	*glabrata*	ATCC 2001
*Candida metapsilosis*	*Candida*	NA	NA	NA	MALDI-TOF at MDL
*Candida parapsilosis*	*Candida*	*parapsilosis*	*Candida*	*parapsilosis*	MALDI-TOF at MDL
*Candida tropicalis*	*Candida*	*tropicalis*	*Candida*	*tropicalis*	CAP B-36-90
*Clavispora lusitaniae*	*Clavispora*	*lusitaniae*	*Clavispora*	*lusitaniae*	CAP F-15-00
*Coccidioides immitis*	*Coccidioides*	*immitis*	*Coccidioides*	*immitis*	Coccidioides real-time PCR at Reference Lab
*Coccidioides immitis*	*Coccidioides*	*immitis*	*Coccidioides*	*immitis*	Coccidioides real-time PCR at Reference Lab
*Cryptococcus gattii VGI*	*Cryptococcus*	*gattii*	*Cryptococcus*	*gattii*	ATCC MYA 4560
*Cryptococcus neoformans*	*Cryptococcus*	*neoformans*	*Cryptococcus*	*neoformans*	ATCC 204092
*Fusarium* sp.	*Fusarium*	NA	*Fusarium*	NA	Morphology
*Kluyveromyces marxianus*	*Kluyveromyces*	*marxianus*	*Kluyveromyces*	NA	ATCC 2512
*Pichia kudriavzevii*	*Pichia*	*kudriavzevii*	*Pichia*	*kudriavzevii*	CAP B-24-92
*Trichophyton mentagrophytes*	NA	NA	NA	NA	ATCC 9533
*Yarrowia lipolytica*	*Yarrowia*	*lipolytica*	*Yarrowia*	*lipolytica*	MALDI-TOF at MDL

Expected genus or species assignments were determined by the reference laboratory using the molecular or biochemical approaches indicated. The NCBI organism name column indicates the known taxonomic information about the sample based on molecular or biochemical approaches. The expected GAMBIT genus assignment and expected gambit species assignment columns indicate the expected taxonomic assignment by GAMBIT based on the representation of that taxon within the GAMBIT database. An “NA” is shown if either the genus or species is missing from the database. The observed GAMBIT genus assignment and observed gambit species assignment columns indicate the actual taxonomic assignment by GAMBIT. An “NA” is shown if GAMBIT did not report an assignment at that taxonomic level.

### 3.5. Clade typing validation

TheiaEuk performs clade typing on genomes that are identified as *C. auris* by GAMBIT using the clade-typer module (Materials and Methods). To validate this functionality, 302 samples with determined clade types from published datasets were compared against the results from TheiaEuk (Table 3) (49). These samples represented four of the five *C. auris* clades (clade I: 126 samples, clade II: 5 samples, clade III: 51 samples, clade IV: 120 samples). All clade assignments made by TheiaEuk were found to match the previously published clade assignments except for one sample which was assigned to clade I despite being previously described as clade III. This genome (strain B16401) was also previously assigned to clade I by another genomic analysis approach, suggesting that the clade identity is controversial for this strain (41). Four samples were not assigned to clades because GAMBIT failed to confidently assign the sample as *C. auris*. Clade typing outcomes for each specimen are available in Supplementary Table S6. TheiaEuk performed accurate clade assignment in 99.6% of cases and therefore enables rapid determination of sample clade without the need for other phylogenetic analysis.

**Table 3:** Clade typing validation using 302 *C. auris* samples spanning four major clades.

	Clade from publication				
Clade-typer results	Total: 302	Clade I	Clade II	Clade III	Clade IV
	Clade I	123	0	1	0
	Clade II	0	5	0	0
	Clade III	0	0	50	0
	Clade IV	0	0	0	119
	Clade-typer skipped	3	0	0	1

Cladetyper results compared to the clades assigned by the original publication (49). The clades assigned by the clade-typer module in TheiaEuk are along the left axis and the clades from the original publication are across the top. Samples that were not successfully assigned to the *C. auris* taxa by GAMBIT were skipped by the clade-typer module. Only one sample produced a discordant result between the clade-typer result (clade I) and the result reported in the original publication (clade III).

### 3.6. Antimicrobial resistance determinant detection validation

TheiaEuk detects mutations in select antimicrobial resistance genes by aligning reads to a *C. auris* clade-specific reference genome and querying the resulting variant-calling output for associated gene and product names. We sought to verify that TheiaEuk reports mutations in genes associated with antimicrobial resistance from genomic data with known mutation status. To this end, three published datasets with genomic data spanning four *C. auris* clades were identified in which presence or absence of *FKS1* and *ERG11* mutations was noted (55–57). The genomic data was analyzed using TheiaEuk and determined that TheiaEuk correctly identified all known mutations in *FKS1* and *ERG11* for 219 samples (Table 4, results from each sample are available in Supplementary Table S7). Because TheiaEuk reports these mutations from variant-calling data, the choice of reference genome impacts the mutations reported by TheiaEuk. It is observed that the default clade III reference genome in TheiaEuk incorporates a known azole resistance mutation: *ERG11* V125A/F126L (56). Likewise, the clade IV reference genome incorporates the *ERG11* Y132F mutation (61).

**Table 4:** TheiaEuk accurately identified mutations in *FKS1* (top) and *ERG11* (bottom) for 219 *C. auris* genomes from published datasets.

		Expected	
		*FKS1* mutation	No *FKS1* mutation
Observed	*FKS1* mutation	44	0
	No *FKS1* mutation	0	175

		Expected	
		*ERG11* mutation	No *ERG11* mutation
Observed	*ERG11* Mutation	161	0
	No *ERG11* Mutation	0	58

Samples spanned four *C. auris* clades: clade I (33 samples), clade II (7 samples), clade III (94 samples), and clade IV (85 samples). The number of expected missense, stop codon, and indel mutations detected in *FKS1* and *ERG11* based on the mutations reported in the original publication was compared to the observed number of mutations in these genes reported by TheiaEuk. The default clade III and IV reference genomes in TheiaEuk include known ERG11 mutations, therefore detection of no variant at that site by TheiaEuk was interpreted as agreement with the original publication.

### 3.7. Implementation of TheiaEuk for the southern Nevada outbreak

Since its development, TheiaEuk has been used to analyze 961 *C. auris* isolates from an ongoing outbreak in southern Nevada. Genomic and phylogenetic analysis of the first 209 samples were reported in Gorzalski et al. (29) and the remaining 752 samples are reported for the first time in this study. These 752 specimens were isolated from samples obtained from either patients presenting with symptoms or through screening of long-term care patients between April 2022 to February 2023. Several medical facilities used the Nevada State Public Health Laboratory for routine screening of *C. auris*. Culturing of all PCR positive samples was attempted with sequencing performed on all culture positive specimens. All samples were identified as *C. auris* by TheiaEuk. Twelve samples were excluded from subsequent analysis due to low genome quality; the remainder were assigned to either clade I (*n* = 157) or clade III (*n* = 583). These data represent an ongoing outbreak; the rapid ability to distinguish which isolates belong to the two major outbreaks and which isolates are part of new introductions based on whole-genome sequencing demonstrates the utility of TheiaEuk as a front-line analysis tool for fungal pathogens.

### 3.8. Detection of antimicrobial resistance determinants in southern Nevada outbreak

**Figure 3:**
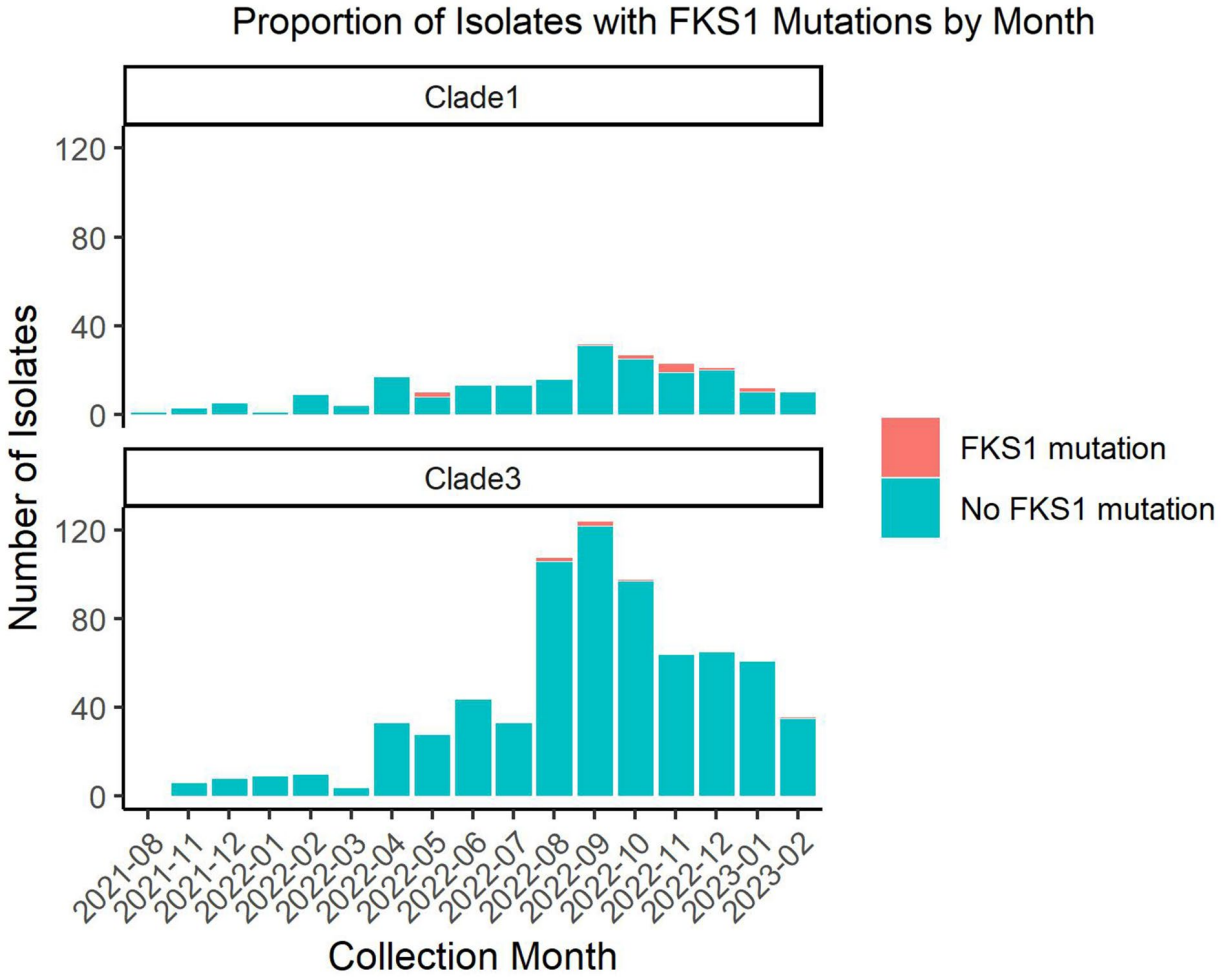
Number of southern Nevada *C. auris* isolates with and without *FKS1* mutations by month. Nine hundred forty-nine *C. auris* isolates from southern Nevada were analyzed using TheiaEuk for presence or absence of *FKS1* mutations. This graph splits the isolates between clade I and clade III representing the two major outbreaks in southern Nevada. The data is represented by month with the number of isolates with the wild-type *FKS1* sequence shown in teal and the number of isolates with a mutant *FKS1* sequence shown in orange. This figure excludes one sample collected in January of 2020 which precedes the ongoing outbreaks.

The TheiaEuk pipeline enables monitoring of mutations in genes associated with echinocandin resistance, particularly *FKS1*. The relevance of this analysis in the southern Nevada outbreak was examined by two methods. Firstly, the accumulation of *FKS1* mutations over time was examined during the outbreak using data from this work and Gorzalski et al. (Figure 3) (29). These mutations occur in strains that share the complete genetic background of non-*FKS1* mutant isolates in the Nevada outbreak. Thus, the most parsimonious explanation for the occurrence of *FKS1* mutations is that they evolved during the outbreak, suggesting that they are in response to the treatment by the frontline antifungals for *C. auris* which are all in the echinocandin class. Mutations in *FKS1* were detected in 18 out of 949 samples throughout the outbreak and were found to represent 7 distinct amino acid substitutions: Ser639Phe, Leu640Val, Arg641Gly, Arg641Ser, Asp642Tyr, Leu686Phe, and Ile1361Thr.

**Figure 4:**
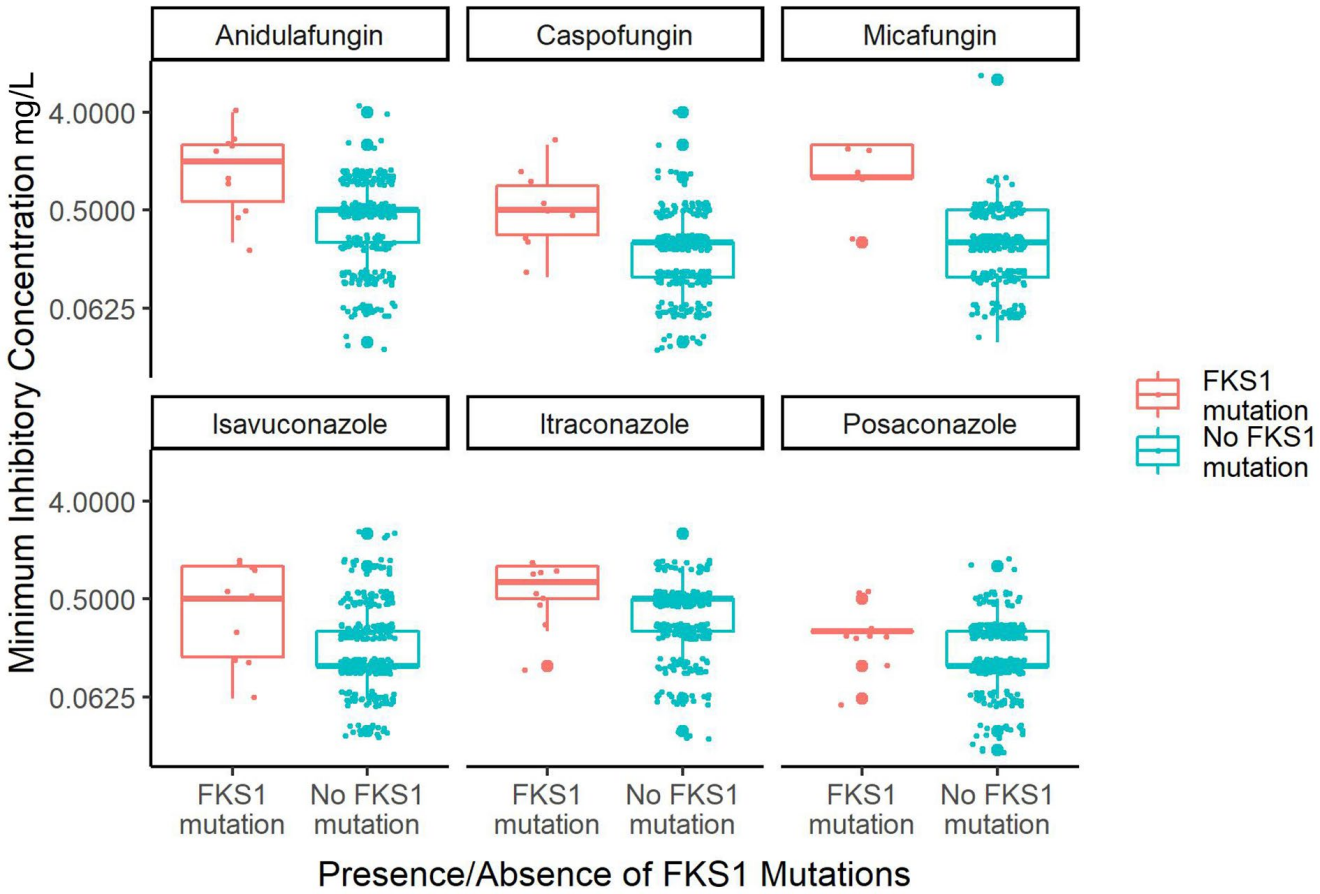
Box plot of MIC data for the six major antifungals that treat *C. auris* based on *FKS1* mutation status. Plotted are the 326 isolates from the southern Nevada *C. auris* outbreak that have MIC data for any of six antifungals. Only 247 isolates have MIC data for micafungin, otherwise *n* = 326 for all other drugs. The orange box plots indicate isolates containing *FKS1* mutations and the teal plots indicate isolates containing *FKS1* wild-type sequence. Small dots indicate individual sample MIC measurements whereas large dots indicate outlying data points of the boxplot.

Secondly, the MIC data for six antifungals that were available for isolates in this dataset were examined. The data was parsed based on presence or absence of *FKS1* mutations (Figure 4). Among the six antifungals, there are three echinocandins: anidulafungin, caspofungin and micafungin. Isolates with *FKS1* mutations exhibit a significantly reduced susceptibility to echinocandins relative to isolates without *FKS1* mutations (Wilcoxon rank sum test with continuity correction: anidulafungin value of *p* = 0.0004511, caspofungin *p*-value = 0.000576, micafungin *p*-value =0.001556). Reduced susceptibility to azoles was also observed for isolates with *FKS1* mutations to a lesser extent and this trend was significant in two drugs (Wilcoxon rank sum test with continuity correction: isavuconazole *p*-value = 0.024270.02203, itraconazole *p*-value = 0.009552, posaconazole *p*-value = 0.05928). While FKS1 mutations were correlated with reduced susceptibility to azoles, it is unlikely that they were responsible for the reduced susceptibility given the distinct mechanisms of action of echinocandins and azoles.

## 4. Discussion

Fungal diseases represent a major threat to public health as evidenced by increasing mortality rates in recent years (62). However, these eukaryotic agents have not incurred the same focus of prokaryotic pathogens, especially in the realm of whole-genome sequence identification and surveillance. This is likely due to the complex nature of their laboratory diagnoses, and the relative paucity of genomic tools to assess them (3–8). The introduction of TheiaEuk provides a platform to utilize whole-genome sequencing of fungal microbial pathogens in both the research and clinical setting.

A novel contribution of this work is the development and assessment of a fungal taxonomic identification process from WGS data. The primary identification engine (GAMBIT) has been utilized in a CLIA regulatory environment to report clinical diagnostic identifications of prokaryotic pathogens (39). Here we extended the same logic to fungal pathogens and laid the groundwork for a similar validation. This is important for clinical laboratories as fungal pathogens often possess complex and ambiguous biochemical profiles that often result in identifications at only the genus level. Moreover, the expertise in mycology to make routine laboratory diagnosis is waning (63). Creating a fungal identification pipeline using whole genome sequencing that will be implemented in a regulatory environment should increase the number of clinically relevant fungal genomes that are produced by public health laboratories and other health care providers (39). This will allow the initial fungal database presented here to be updated and extended to additional fungal species, thus increasing impact.

The regular incorporation of whole genome sequencing to fungal pathogen surveillance provides not only robust taxonomic identification but additional insights regarding genetic relatedness. For example, the use of TheiaEuk in the ongoing *C. auris* outbreak in southern Nevada demonstrated that specimens collected during the same time period represented distinct introductions because it revealed that samples were from two different clades. Also, while the TheiaEuk pipeline does not directly produce phylogenetic trees from specimen sets, the output files generated by the workflow are compatible with numerous downstream tools for more granular phylogenetic analysis. Examples include the kSNP3 workflow and MashTree workflow, both of which are open source and available for analysis using Terra (30, 64, 65). Through these subsequent analyses, transmission networks among fungal pathogens may be discerned.

Examination of the southern Nevada *C. auris* outbreak by TheiaEuk also reveals the necessity of pipelines like the one described for detection of antimicrobial resistance determinants. Currently, there are three classes of antifungals that can treat *C. auris*. Yet, most *C. auris* strains (93%) are resistant to fluconazole, and another 35% are resistant to AmpB (13). This leaves echinocandins as the major frontline defense to *C. auris*. Given that *C. auris* forms biofilms on both biotic and abiotic surfaces, exists asymptomatically on colonized patients, carries drug resistance, and poses potential lethal consequences upon septic infection, *C. auris* presents a real threat to our health care system (66). This threat is amplified if echinocandin resistant isolates become endemic to communities. The ability to detect and to take disease control action on isolates of *C. auris* that have mutations in *FKS1* that correlate with decreased susceptibility to echinocandins is critical to mitigate this new threat. Unfortunately, current phenotype-based systems that assess for decreased susceptibility rely on centralized services where isolates of interest are sent, cultured, then grown and tested against a series of antifungals. This is followed by the reporting of data in a systematic form which often results in a considerable turnaround time to inform health care providers that they have a resistant or decreased susceptibility isolate of *C. auris*. This lag may prevent the most effective actions from being taken to control these potential threat organisms. Whole-genome sequencing and the detection of *FKS1* mutations decrease this timeline significantly and provide a method for disease control investigators to stay ahead of echinocandin resistant strains of *C. auris*.

An often overlooked but increasingly important aspect of bioinformatics tools is the need to be accessible to the broader scientific community, not just bioinformaticians. Innovative tools conceived and developed within the disease pillars of academic and government laboratories are often inaccessible to the average public health scientist with no training, experience, or resources in command line bioinformatics. To this end, we share the same philosophy as Black et al. in their recommendations for supporting open pathogen genomic analysis in public health (67). TheiaEuk was intentionally developed from the beginning to be (1) reproducible in the way it implements containerization, versioning, workflow management, and auditability, (2) scalable in the utilization of cloud resources, and (3) deployable within hours using the open bioinformatics platform Terra for workflow registry and web portal accessibility. This open bioinformatics platform will then bridge across all disease pillars, where specialty tools designed by disease experts will be accessed and utilized in a common, open environment. This is particularly important for public health laboratories whose pathogen genomic outbreak investigations cover the full spectrum of human and animal pathogens. In addition to accessibility the ability to validate workflows for public health use is vital, something not often encountered in research environments but critical for our public health system. Here, again, the use of the open bioinformatics platform Terra, with the ability to version, audit, and validate every workflow, meets the needs of public health scientists, both nationally and internationally.

## Data availability statement

The original contributions presented in the study are included in the article/Supplementary material, further inquiries can be directed to the corresponding authors.

## Author contributions

FA and MS created bioinformatics pipelines, performed analysis, wrote sections of the manuscript, and helped in revisions. SW, JO, and ED created bioinformatics pipelines and helped in revisions. AG generated data for the paper, performed analysis, wrote sections of the manuscript, and helped in revisions. DS, SK, CH, ES, and VV generated data for the paper and helped in revisions. KL supported and funded the creation of bioinformatic pipelines, created bioinformatics pipelines, and helped in revisions. MP conceived of the projects, performed analysis, wrote sections of the manuscript, and helped in revisions. JS supported and funded the creation of bioinformatic pipelines, wrote sections of the manuscript, and helped in revisions. DH conceived of the projects, generated data, performed analysis, wrote sections of the manuscript, and helped in revisions. All authors contributed to the article and approved the submitted version.

## Funding

This publication was supported by the Nevada State Department of Health and Human Services through Grant Number NU50CK000560 from the Centers for Disease Control and Prevention. Its contents are solely the responsibility of the authors and do not necessarily represent the official views of the Department nor the Centers for Disease Control and Prevention.

## Conflict of interest

FA, MS, SW, JO, ED, KL, and JS were employed by Theiagen Genomics.

The remaining authors declare that the research was conducted in the absence of any commercial or financial relationships that could be construed as a potential conflict of interest.

## Publisher’s note

All claims expressed in this article are solely those of the authors and do not necessarily represent those of their affiliated organizations, or those of the publisher, the editors and the reviewers. Any product that may be evaluated in this article, or claim that may be made by its manufacturer, is not guaranteed or endorsed by the publisher.

## Footnotes

^2^ https://github.com/theiagen/public_health_bioinformatics/blob/PHB-v0.1.0-theiaeuk-manuscript/workflows/standalone_modules/wf_gambit_query.wdl

^3^ https://github.com/theiagen/public_health_bioinformatics/blob/PHB-v0.1.0-theiaeuk-manuscript/workflows/utilities/data_import/wf_sra_fetch.wdl
