# Supplementary material for: TheiaEuk: a species-agnostic bioinformatics workflow for fungal genomic characterization
Source: Front Public Health. 2023 Aug 1;11:1198213. doi: 10.3389/fpubh.2023.1198213 (PMC10428623; doi:10.3389/fpubh.2023.1198213)
Supplement: Supplementary file 1 [file Data_Sheet_1.docx]

***Supplementary Material***

**TheiaEuk: A Species-Agnostic Bioinformatics Workflow for Fungal Genomic Characterization**

**Frank J. Ambrosio III^1┼^, Michelle R. Scribner^1┼^, Sage M. Wright^1^, James R. Otieno^1^, Emma L. Doughty^1^, Andrew Gorzalski^2^, Danielle Denise Siao^2^, Steve Killian^3^, Chi Hau^4^, Emily Schneider^4^, Vici Varghese^3^, Kevin G. Libuit^1^, Mark Pandori^2,5,6*^, Joel R. Sevinsky^1&^, and David Hess^2,5&*^**

^1^ Theiagen Consulting LLC, Highlands Ranch, CO, USA.

^2^ Nevada State Public Health Laboratory, Reno, NV, USA.

^3^ Alameda County Public Health Laboratory, Oakland, CA 94605, USA.

^4^ Public Health Laboratories, Division of Disease Control and Health Statistics, Washington State Department of Health, Shoreline, WA, USA.

^5^ Department of Pathology and Laboratory Medicine, University of Nevada, Reno School of Medicine, Reno, NV, USA.

^6^ Department of Microbiology and Immunology, University of Nevada, Reno School of Medicine, Reno, NV, USA.

^┼^ These authors contributed equally to this work.

^&^ These authors contributed equally to this work.

# Supplementary Tables

**Supplementary Table 1. Default reference genomes used for clade typing and antimicrobial resistance gene detection of *C. auris*.**

| Clade | Genome Accession | Assembly Name | Strain | NCBI Submitter | Included mutations in AMR genes (FKS1,ERG11,FUR1) |
| --- | --- | --- | --- | --- | --- |
| *Candida auris* Clade I | GCA_002759435.2 | Cand_auris_B8441_V2 | B8441 | Centers for Disease Control and Prevention |  |
| *Candida auris* Clade II | GCA_003013715.2 | ASM301371v2 | B11220 | Centers for Disease Control and Prevention |  |
| *Candida auris* Clade III | GCA_002775015.1 | Cand_auris_B11221_V1 | B11221 | Centers for Disease Control and Prevention | ERG11 V125A/F126L |
| *Candida auris* Clade IV | GCA_003014415.1 | Cand_auris_B11243 | B11243 | Centers for Disease Control and Prevention | ERG11 Y132F |
| *Candida auris* Clade V | GCA_016809505.1 | ASM1680950v1 | IFRC2087 | Centers for Disease Control and Prevention |  |

**Supplementary Table 2. Taxonomic groups represented in the GAMBIT fungal database**. Species represented in the GAMBIT fungal database are shown with the calculated species diameter and number of genomes representing that species. The NCBI taxonomy ID for each species is indicated. Unless otherwise indicated, the GAMBIT database uses the species and genus names assigned to a genome by NCBI.

**Supplementary Table 3. NCBI SRA accessions for whole genome sequencing data for fungal specimens from Alameda County Public Health Laboratory.** Specimens were sequenced by the Nevada State Public Health Laboratory.

**Supplementary Table 4. NCBI SRA accessions for whole genome sequencing data for *C. auris* specimens sequenced by the Nevada State Public Health Laboratory.**

**Supplementary Table 5. GAMBIT taxonomic assignments for ATCC mycotic genome collection.**

**Supplementary Table 6. TheiaEuk clade typing results for isolates previously described by Chow et al. (**[**https://doi.org/10.1128/mBio.03364-19**](https://doi.org/10.1128/mBio.03364-19)**).** The originating study which published whole genome sequencing data for the isolate is indicated in “Originating Study” with the associated SRA accession. The clade assigned by Chow et al is indicated in “Published Clade”. The results from TheiaEuk including clade, taxonomic assignment from GAMBIT, and assembly length from Quast are in “TheiaEuk clade type”, “TheiaEuk gambit_predicted_taxon”, and “TheiaEuk assembly_length”, respectively.

**Supplementary Table 7. Mutations detected in antimicrobial resistance-associated genes (*FKS1* and *ERG11*) for genomes with known variant status.** The reference genomes used for variant calling analysis in TheiaEuk differ from those used in the original publications. As a result, mutations listed in the original publications are included in TheiaEuk's reference genomes in some instances and are responsible for any differences shown below.
